# Challenges with interviewer administration of EQ-5D questionnaires in a large-scale population survey in China: a qualitative analysis

**DOI:** 10.1007/s11136-026-04351-4

**Published:** 2026-07-30

**Authors:** Tianxin Pan, Nan Luo, Zhuxin Mao, Xin Hong, Chao Li, Jie Wu, Nancy Devlin

**Affiliations:** 1 Melbourne School of Population and Global Health, University of Melbourne, Melbourne, Australia; 2 Saw Swee Hock School of Public Health, National University of Singapore, Singapore, Singapore; 3 Centre for Health Economics Research and Modelling Infectious Diseases (CHERMID), University of Antwerp, Antwerp, Belgium; 4 Nanjing Municipal Center for Disease Control and Prevention, Nanjing, China; 5 Real World Solutions, IQVIA, Shanghai, China

**Keywords:** EQ-5D, Interviewer effects, Mode of administration, Ceiling effects, Health-related quality of life, China

## Abstract

**Introduction:**

While most adult measures of health-related quality of life (HRQoL) are designed for self-completion, their use in low- and middle-income countries involves interviewer administration. Large-scale population surveys in China that collect EQ-5D data via face-to-face interviews typically show high ceiling effects. We aimed to identify non-standard interviewing approaches in the collection of EQ-5D data in a large-scale population health survey in China and to explore reasons for these non-standard approaches and plausible mechanisms through which these deviations may influence EQ-5D response patterns.

**Methods:**

A random sample of routinely recorded interviews from population health surveys in Nanjing, China were thematically analysed to identify interviewing approaches to collecting EQ-5D-3L. Focus group discussions with ten interviewers were used to explore the reasons for those approaches.

**Results:**

Six non-standard interviewing approaches were identified: skipping questions; altering question wording; combining multiple dimensions into one question; altering response options; selecting responses based on interviewer interpretation of respondent’s narrative; and not asking the interviewee any questions. Three main reasons motivated these non-standard approaches: (a) comprehension and response challenges among low-literacy respondents; (b) survey structure, including overall length, positioning of EQ-5D questions and respondent burden leading to time-saving interview strategies; (c) limited interviewer knowledge of self-reported health measures, including a tendency to view EQ-5D as an observer-assessed instrument.

**Conclusion:**

Our findings highlight challenges in interviewer administration of the EQ-5D in large-scale population surveys. The non-standard approaches observed may affect data quality, potentially contribute to ceiling effects, and complicate the interpretation of these data being ‘self-reported’. Providing training on EQ-5D data collection to interviewers may be a first step to strengthening data collection processes.

**Supplementary Information:**

The online version contains supplementary material available at https://doi.org/10.1007/s11136-026-04351-4.

## Introduction

EQ-5D instruments have been widely used for measuring health-related quality of life (HRQoL) in general populations across many countries [1]. While HRQoL measures are designed to capture self-reported health, and are self-completed in many contexts, interviewer administration is common in low- and middle-income countries (LMICs) [2, 3]. In China, large-scale population surveys typically collect data via face-to-face interviews (e.g. National Health Service Surveys [4], Survey of Health Index of Chinese Families [5]). However, studies from China have reported high ceiling effects in EQ-5D data collected in these surveys (i.e., reporting no problems on all dimensions): over 80% for the EQ-5D-3L [4, 6, 7] and over 60% for the EQ-5D-5L [5, 8], which are higher than those reported in many other countries [2].

Several potential reasons have been proposed to explain the high ceiling effects observed in China, including (1) cultural-specific concepts of health [4, 9–11]; (2) item wording and phrasing [6, 12]; (3) response-scale heterogeneity [13], and (4) data collection and implementation, including using interviewer-led administration, which is common in China [4]. For example, the National Health Service Survey, conducted at the national level, often involves lengthy questionnaires covering many aspects of health service use, health conditions and health-seeking behaviour, and can involve up to 2000 interviewers across different regions [4]. Studies have hypothesised that the mode of administration (MOA) and implementation of data collection can contribute to the high ceiling effect [4]. However, no empirical evidence exists on whether and how face-to-face, interviewer-led mode may influence the distribution of EQ-5D data in general population surveys in China. This study explored, this issue by analysing audio recordings of interviewer-administered EQ-5D questions from a municipal-level large-scale health survey in China and conducting focus group discussions with a sample of survey interviewers. The specific aims were to: (1) identify and describe non-standard interviewing approaches in the collection of EQ-5D profile and EQ VAS data, within the context of a large-scale population health survey in China; and (2) explore reasons for these non-standard approaches and plausible mechanisms through which these deviations may influence EQ-5D response patterns.

## Methods

This study comprised two components: (1) a thematic analysis of EQ-5D interview recordings from a municipal-level population health survey in China to identify interviewing approaches; (2) Focus Group (FG) discussions with survey interviewers to explore reasons for these approaches. We reported methods following the Consolidated criteria for reporting qualitative research (COREQ) [14].

### Setting

The health survey was conducted in Nanjing, China by the Nanjing Centre for Disease Control and Prevention (Nanjing CDC). Since 2020, the survey has been conducted annually in 2–3 of Nanjing’s 11 districts, using a multistage (subdistrict, community, and household), stratified cluster random sampling strategy within each district. One member from each selected household was randomly chosen to participate. In Nanjing, districts comprise 6–13 subdistricts, each containing 6–12 communities, with each subdistrict served by 1–3 primary healthcare centres (PHCs).

Participants were invited to local PHC for a health check-up followed by a tablet-based face-to-face interview. Trained interviewers were local PHC general practitioners or officers who had a medical or public health background and had worked at least one year at the PHC. Standardised training was conducted by Nanjing CDC at each PHC prior to fieldwork, focusing on the survey site set up including the organisation of health check-ups, tablet use, data management, questionnaire content and on-site interview skills. No specific training on EQ-5D administration was provided. Interviewers were instructed to follow the questionnaire verbatim, using local dialects only when necessary and without altering the wording or structure. In total, 398 interviewers conducted the fieldwork in 2020. All interviews were audio recorded for quality control. The 2020 survey included 29,503 respondents at 23 PHCs from three districts; the 2021 survey had 23,180 respondents from two districts.

The questionnaire included the following components: socio-demographic questions (10 items, e.g. age, gender, occupation, health insurance, household income), health-related behaviours (7 items on smoking, 13 on alcohol and tea consumption, 16 on diet, 20 on physical activities, and 7 on sleep), self-reported chronic conditions and family history (40 items), health service use (3 items), health knowledge (10 items), dental health (10 items), and the EQ-5D questionnaire (6 items). Following the EQ-5D, additional modules were administered based on age: the SF-12 (12 items) and a COPD knowledge and respiratory symptoms module (39 items) for participants aged ≥ 40 years, and the Mini-Mental State Examination (11 items) for those aged ≥ 65 years.

The EQ-5D comprises a descriptive system and the EQ VAS, a visual analogue scale (0 = worst imaginable health to 100 = best imaginable health). The descriptive system contains five dimensions: Mobility (MO), Self-care (SC), Usual Activity (UA), Pain/Discomfort (PD), and Anxiety/Depression (AD) [15]. The self-completion EQ-5D-3L version was used in 2020, and the self-completion EQ-5D-5L version in 2021. In other words, both surveys used non-IA versions and were administered by interviewers.

### Thematic analysis of survey recordings

Nanjing CDC provided access to all EQ-5D-3L interview audio recordings. We randomly selected two interviews from all available recordings from each of the 23 PHCs in the 2020 survey (n = 46 recordings). For each PHC, respondents were assigned sequential IDs, and two IDs were randomly generated using Excel’s RANDBETWEEN function within the corresponding range. The recordings associated with these IDs were selected for analysis. Based on previous field observations, we anticipated data saturation by approximately 46 recordings. Additional recordings would have been analysed if new themes continued to emerge.

Thematic analysis was used without a pre-existing framework. All audio recordings were transcribed for coding by TP, a native speaker of the Nanjing dialect. The Nanjing dialect is broadly similar to standard Mandarin (Putonghua) and can be understood by Mandarin speakers. Two researchers (TP, ZM) independently coded 50% of the recordings (n = 24), identifying non-standard interviewing approaches. Coding was conducted iteratively, with categories refined after each round of 12 recordings. Both researchers had received training in qualitative research methods and had experience in conducting qualitative interviews and analysis. According to the official interviewer-administered (IA) version [16], interviewers should read each question and all response options verbatim and ask respondents to choose their response. If respondents struggle, interviewers should repeat the question verbatim and ask the respondent to answer in a way that most closely resembles their thoughts about their health today. Any deviations from this process were considered non-standard. Four researchers (TP, ZM, NL, ND) reviewed the coding and categorisation to ensure consistency and triangulation. Recordings were analysed sequentially by PHC, with two recordings from each centre. Data saturation was reached after coding 24 recordings (i.e., within the first 12 centres); however, all 46 recordings were analysed. The remaining recordings (n = 22) were coded by TP and reviewed by ZM.

### Focus groups with survey-interviewers

We conducted two FG discussions in February 2022, each with five survey-interviewers (local PHC doctors) recruited from different PHC centres from one of the two districts participating in the 2021 survey. Discussions were conducted using a hybrid model: Two study team members (XH, JW) and five survey-interviewers met in a district CDC meeting room in Nanjing, while other team members joined via Zoom. A district CDC officer who facilitated logistics attended the introductory session at the beginning of the discussion then left the room.

Based on findings from analysis of recordings and literature [12, 17, 18], four researchers (TP, ZM, NL, ND) developed a topic guide and prompts (see electronic supplementary material 1 [ESM]).

The focus groups comprised two sessions. Session one explored: (1) how survey-interviewers administered EQ-5D-5L questions in the 2021 survey; (2) reasons for using those approaches; (3) challenges encountered in the interview regarding the EQ-5D-5L questions. We were aware that self-completion versions of the EQ-5D were used in Nanjing CDC surveys and that survey-interviewers had not seen the IA version and notes. Therefore, session two focused on the potential use of the IA version. Specifically, session two covered: (1) survey-interviewers’ views on the IA version and preference between IA and the ‘self-completion’ versions in population health surveys; (2) the feasibility of implementing the IA version; and (3) suggestions for improving EQ-5D data collection in large-scale population health surveys.

Discussions were recorded and notes taken. Data were analysed using thematic analysis with a manual coding approach in Microsoft Word. Transcripts were reviewed, annotated and coded iteratively through repeated reading, focusing on reasons for and potential solutions to non-standard interviewing approaches. Codes were refined through team discussion (all authors). Further details on the focus groups are provided in ESM1.

Ethical approval was granted by Nanjing CDC Ethics Committee (PJ2020-B001-01 on 17th of August 2020, PJ2022-A001-04 on 10th of February 2022).

## Results

### Sample characteristics

Characteristics of the randomly selected recording sample (n = 46) are presented in Table S1 (ESM2). The mean age was 53 years (SD 13), 58.7% were female, and 28.3% had no formal education or only primary education. A total of 45 interviewers were represented in the 46 recordings. The ceiling effect of EQ-5D-3L in the recording sample was 71.7%, and the mean (SD) EQ VAS score was 75.3 (14.9). In the full 2020 sample (n = 29,503), the mean age was 50 years (SD 15), 54.0% were female, and 30.8% had no formal education or only primary education. The ceiling effect was 79.6%, and the mean EQ VAS score was 79.4 (SD 13.9).

Among the ten FG participants (interviewers for 2021 survey), two were men and eight were women. In the 2021 full sample, the ceiling effects of EQ-5D-5L were 71.1%, mean EQ VAS score was 81.8 (SD 12.1).

### Non-standard interviewer approaches identified

Analysis of 46 EQ-5D-3L recordings identified six non-standard approaches: (1) skipping asking some questions (58.7%), where interviewers did not verbally ask the question but still recorded responses on behalf of the respondent, particularly for mobility, self-care and usual activities; (2) altering question wording (73.9%), e.g. asking leading questions or providing interviewers’ own interpretations of dimensions; (3) combining multiple dimensions into a single question (26.1%), which usually involves altering the wording of the questions; (4) altering response options (39.1%), particularly transforming the responses to binary options (having any problems or no problems); (5) choosing responses based on interviewers’ own interpretation of the respondents’ narratives (30.4%); and (6) not asking the interviewee any questions from the EQ-5D descriptive system (10.9%). In addition, six recordings (13.0%) containing no EQ-5D conversation were identified as cases where respondents completed the questionnaire via self-completion, based on review of other parts of the recordings. Table 1 presents these approaches, with explanations and examples. We did not identify any examples of interviews fully adhered to the expected ways of collecting EQ-5D data via interviewer-administered mode.

**Table 1:** Non-standard interviewing approaches to administering the EQ-5D descriptive system and EQ VAS and examples

Non-standard interview approach identified	Explanation	Example
(1) Skipping asking some questions	Not reading out or asking each of the five questions. Mobility, self-care and usual activities were most frequently skipped dimensions	*“(Silence for 5 s)…. have you ever experienced any anxiety or depression?” [Recording sample 10]*
(2) Altering question wording	Asking leading questions; Using made-up examples or providing interviewer’s own interpretations of dimensions; Asking only part of the composite dimensions (i.e., Pain/discomfort, Anxiety/Depression)	*“You don’t have any anxiety or depression, right?” [Recording sample 12]* *“Do you have emotional anxiety, like lacking interest in doing things?” [Recording sample 9]* *“Do you have any pain?” [Recording sample 6]*
(3) Combining multiple dimensions into one question	Asking several dimensions within a single question, often involving changes to wording	*“You don’t have any problems in walking about, taking care of yourself, without any difficulties, and you can do your everyday work, study and housework, right? [Recording sample 21]”* *“All good about your physical function? [Recording sample 5]”*
(4) Altering the response options	Changing the wording of response levels. In particular, transforming the responses to a binary option (having any problems/no problems), is very common in the recordings	*“(Do you have) any pain or discomfort?… which type of pain, feel sore?” [Recording sample 24]* *“Do you have any pain?” [Recording sample 6]*
(5) Choosing responses based on interviewers’ own interpretation of respondents’ narrative	As the interviewer altering the wording of the questions and response options, respondents would provide narrative answers. The interviewer then selects a response based on their own interpretation and judgement	*“Interviewer: Do you feel nervous or anxious?”* *“Respondent: Yes”* *“Interviewer: moderate or severe?”* *“Respondent: More than moderate”* *[Recording sample 21; level 2 was selected for this dimension]*
(6) Not asking the interviewee any questions	Completing the questionnaire for the respondent based on interviewer observation only, without asking any questions*	*Not Applicable [Recording sample 3]*

These examples of responses in the audio recordings were in Chinese and were translated by TP and checked by ZM

^*^This does not include cases where respondents completed the EQ-5D via self-completion

Multiple non-standard approaches often occurred within a single interview (see Box 1 for an example). For the EQ VAS, the most common issues were: (1) altering question wording, including leading questions; and (2) marking scores based on the interviewer’s interpretation of a respondent’s answer. [BOX 1]

### Reasons underlying non-standard interviewer approaches

**Fig. 1:**
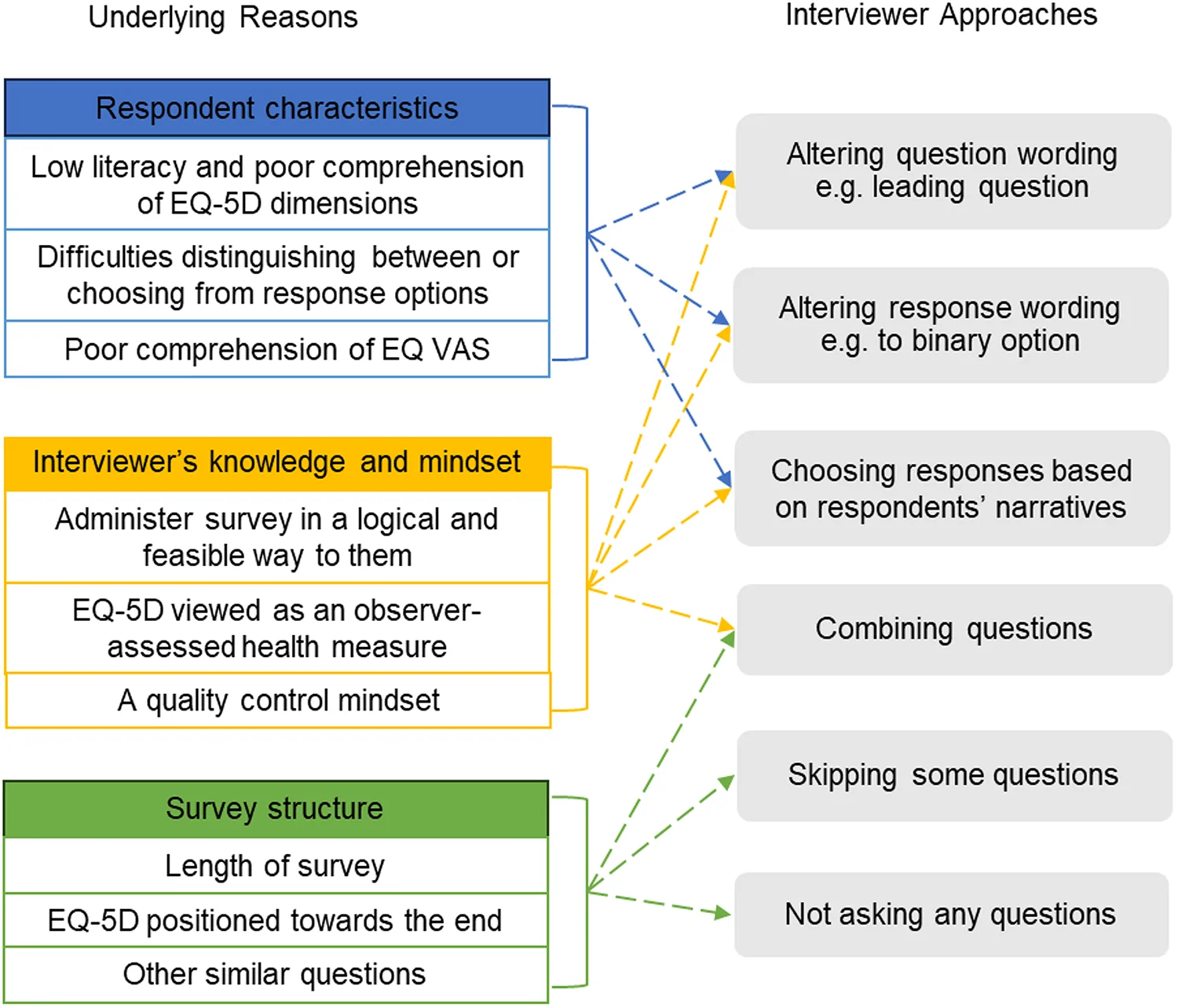
Underlying reasons (respondent characteristics, survey structure, and interviewer factors) and their potential links to non-standard interviewer approaches

Three principal themes and eight sub-themes emerged from the focus group discussions (Fig. 1). Illustrative quotes are provided in Table 2.

**Box 1:** Examples of non-standard interview approaches

* “Interviewer: So, you can take care of yourself, do your usual activity, have no discomfort, and no anxiety. Your health today is about 90?*
*Respondent*: *More or less”*
***[Recording Sample 1. The recorded profile and VAS was 11111 and 90 for this respondent]***
“(Silence for 5s) *Interviewer: Hmm…* have you ever experienced any anxiety or depression?
*Respondent*: No.
*Interviewer*: Not feeling anxious, and no pain?
*Respondent*: I can feel pain.
*Interviewer*: Where about?
*Respondent*: On my legs.
*Interviewer*: So that’s moderate pain?
*Respondent*: Today I feel alright.
*Interviewer*: Okay then I will choose moderate pain for you.
*Respondent*: Yes, moderate, just pain on my leg. I cannot even switch on the air conditioners.
*Interviewer*: From 0 to 100, how would you rate your health?
*Respondent*: I feel… all good?
*Respondent*: Hmm… from 0 to 100...
*Interviewer*: 80? Out of 100
*Interviewer*: 80?
*Respondent*: Okay, 80! I feel alright
***[Recording Sample 10. The recorded profile and VAS was 11121 (on 3L) and 80 for this respondent]***
*“Interviewer*: How good or bad do you feel about your health?
*Respondent:* Good.
*Interviewer*: If it is “good”, I will choose something between 60 and 80”
***Recording Sample 23. The recorded VAS was 70 for this respondent]***

**Table 2:** Reasons contributing to ‘non-standard’ interviewer approaches and quotes from focus group discussions

Theme 1 Respondent characteristics: comprehension and response challenges among low-literacy respondents
**Sub-theme 1.1 Poor comprehension of EQ-5D dimensions**
*“I usually ask these questions in a more specific way. For example, for walking about, I would ask whether they have problems in climbing stairs, or whether they can walk 100 m without break. Based on the respondent’s health status, comprehension and answers to previous questions in the survey, I adapt the questions in a way that can be understood by the respondents. “(focus group participant 1)* *“For anxiety and depression, I might ask about their sleep quality, whether they dream a lot. Or I would ask if there is anything in the household that worries them, or anything they are unhappy about. It’s like a chit chat.” (focus group participant 10)*
**Sub-theme 1.2 Difficulties distinguishing between or choosing from EQ-5D response options**
*“Among the five questions, respondents usually need our help to interpret and choose for anxiety/depression dimension. The main challenge for them is quantifying the level of problems. They usually say, ‘I sometimes feel unhappy’ but what is the response level for ‘sometimes’…” (focus group participant 6)* *“If older adults reported having problems, I would usually select one or two response options that I think is more relevant to them and ask them which level. I wouldn’t read out all the options.” (focus group participant 8)* *“To most people, severe problems in walking about basically means needing a wheelchair or being confined to bed. It is the same.” (focus group participant 7)*
**Sub-theme 1.3 Poor comprehension of the concept of EQ VAS**
*“It is very common that when we ask older adults to mark their health today, they would say they don’t know what score to choose and ask what we think.” (focus group participant 8)* *“Most of them felt they cannot mark a score for their health. They didn’t have any reference or anchor. Some may feel their health is ‘alright’ or ‘not bad’ but cannot map it to a score. It could be 80 or 90, but what makes the 10-points difference” (focus group participant 4)*
Theme 2 Survey structure: length, positioning, and respondent burden leading to time-saving interview strategies
**Sub-theme 2.1 EQ-5D positioned towards the end of a long survey and respondents become impatient and bored**
*“People became very impatient at the end of the survey. Some just wanted to finish quickly, so they would say, "I don’t have any problems". As a survey interviewer, we couldn’t intervene too much, so we recorded what they said.” (focus group participant 3)* *“After (doing or waiting for) a few interviews, both interviewers and respondents became tired and impatient. It is very natural for us to first ask whether they have any problems then what level of problems, to speed up the process” (focus group participant 9)*
**Sub-theme 2.2 Inferring answers to EQ-5D questions from earlier questions**
*“For mobility—We had already asked about physical activity in previous parts – whether they exercise, light or moderate *etc*. So we basically knew whether respondents had problems in walking.” (focus group participant 2)*
Theme 3 Interviewer knowledge, mindset and practices: limited knowledge about the EQ-5D and the self-reported health principles
**Sub-theme 3.1 EQ-5D viewed as an observer-assessed health measure**
*“For example, for self-care or mobility, most participants walked to our centre and did not use a wheelchair, so it is easy for us to judge. The only thing we need to ask or explain is anxiety. Mental health problems have received more attention so sometimes the respondents could answer by themselves. This is a subjective question so we cannot intervene their choice too much.” (focus group participant 2)*
**Sub-theme 3.2 A “Quality control” mindset**
*“For example, if someone said they sprained their foot in an earlier section but reported no pain, I would remind them, or ask ‘Are you sure there is no pain’” (focus group participant 2)* *“There are circumstances that I think the respondent has generally good health but they gave a low VAS score. I would double check or ask why, but ultimately would record what they scored.” (focus group participant 9)*
**Sub-theme 3.3 Interviewers invite responses in a manner that is most logical and feasible to them**
*“I think it is more logical to ask in two steps – whether they have problems and the level of problems. If we start by asking about the level, it feels like we are assuming they have problems” (focus group participant 5)* *“Sometimes I would change the order. For example, someone sprained the ankle and said slight pain. Then I would ask mobility and pain together. We need to be more flexible” (focus group participant 6)*

Quotes were translated from Chinese into English and lightly edited for clarity without altering meaning

#### Theme 1 Respondent characteristics: comprehension and response challenges among low-literacy respondents

- Sub-theme 1.1 Low literacy and poor comprehension of EQ-5D dimensions

Survey-interviewers reported that they frequently adapted questions to improve respondent understanding, based on their observations of the respondent and prior questionnaire responses. Respondents experiencing difficulties were typically older adults (≥ 60 years), from rural areas and with low literacy. Survey-interviewers often used made-up examples of the problems in each dimension and asked these questions in a conversation-like style.

For mobility, self-care and usual activities, examples included climbing stairs and whether the respondents walked to the PHC on the day, dressing with or without help, and doing housework. For pain/discomfort, survey-interviewers tended to focus on the pain, sometimes omitting the ‘discomfort’ component. Low-literacy respondents were reported to have limited understanding of the terms ‘anxiety’ and ‘depression’, therefore survey-interviewers used terms such as ‘unhappy’, ‘worry’, and ‘sad’.

- Sub-theme 1.2 Difficulties distinguishing between or choosing from EQ-5D response options

Survey-interviewers reported that older and less-educated respondents struggled to distinguish between levels of problems, particularly levels 2–4 (slight, moderate, severe) and to select an appropriate response. Some respondents asked for clarification of level meanings, noting that the level labels were not commonly used in everyday language in their survey districts. To address this, survey-interviewers often used alternative wording or specific examples. Furthermore, FG participants felt that respondents struggled to ‘quantify’ the levels of problems, i.e., ‘mapping’ their personal situations onto the available response options. As a result, survey-interviewers sometimes selected the response option based on their interpretation of respondents’ narratives. Among the five dimensions, pain/discomfort and anxiety/depression were reported to be most difficult. Two FG participants also indicated that the difference between Levels 4 and 5 in MO and SC was unclear.

- Sub-theme 1.3 Poor comprehension of the concept of EQ VAS

All FG participants reported that respondents had difficulty understanding and completing EQ VAS. It was described as “very subjective”, with respondents had “no idea” what score to place. Some respondents asked the interviewer to choose for them, while survey-interviewers sometimes suggested a range or specific value. FG participants pointed out that, because respondent data were entered via a tablet and some respondents were unable to read, the EQ VAS scale was not always shown to respondents.

#### Theme 2 Survey structure: length, positioning, and respondent burden leading to time-saving interview strategies

- Sub-theme 2.1 EQ-5D is positioned towards the end in a lengthy survey and respondents become impatient and bored.

FG participants reported that the full survey took 30–40 min. By the time respondents reached the EQ-5D section, they were impatient and fatigued, or aware that other respondents were waiting. As a result, both survey-interviewers and respondents tended to rush through the EQ-5D questions.

- Sub-theme 2.2 Inferring answers to EQ-5D questions from earlier questions

Survey-interviewers perceived some EQ-5D questions as overlapping with earlier survey questions (e.g., physical activity, health history). Respondents sometimes became confused or frustrated by repeated questions. To manage this, interviewers occasionally skipped some of the EQ-5D questions, inferring answers from the respondent’s earlier responses. This behaviour was more common for functioning dimensions (MO, SC, UA) than symptom dimensions (PD and AD).

#### Theme 3 Interviewer knowledge, mindset and practices: limited knowledge about the EQ-5D and self-reported health principles

Survey interviewers received general survey training but no EQ-5D-specific training. It was clear, from our focus group discussions, that interviewers demonstrated limited knowledge of the EQ-5D and the principles of self-reported health measures and deviated from the approach to data collection intended by the instrument developers.

- Sub-theme 3.1 EQ-5D viewed as an observer-assessed health measure

Although not directly raised by FG participants, we found that terms such as ‘observing’ and ‘judging’ problems appeared frequently during FG discussion. When FG participants described respondents’ difficulties in responding to the EQ-5D questionnaire, the terms ‘subjective’ (in the pejorative sense) or ‘abstract’ were used. Most survey interviewers were doctors, and their views appeared influenced by their clinical perspective. They tended to view EQ-5D as an observer-assessed or ‘clinical’ measure, rather than a measure of patients’ self-reported health, and felt responsible for evaluating respondents’ health status. One FG participant asked us whether the EQ-5D had a “standard” to measure the level of problems or was totally subjective to respondents to report.

- Sub-theme 3.2 A “quality control” mindset

Some FG participants shared that they usually formed their own judgement of the respondents’ HRQoL before respondents answered EQ-5D questions. When responses differed from their expectations, they prompted respondents by referring to relevant health issues mentioned earlier in the survey.

- Sub-theme 3.3 Interviewers invite responses in a manner that is most logical and feasible to them.

Asking the descriptive system questions in two steps was mentioned as a very common approach. Survey interviewers usually first asked whether any problems existed, and then, if the response was positive, would ask about the level of severity. Survey-interviewers considered this more logical and efficient. In addition, some FG participants reported changing the order of EQ-5D dimensions.

### Interviewers’ view on the official IA version and its feasibility in large-scale population surveys

#### Advantages of IA

FG participants preferred the IA wording for the descriptive system, noting the use of second-person pronouns and standardised guidance [16].

#### Barriers to using IA

Despite these advantages, all FG participants felt that strictly following IA guidance for EQ-5D was not feasible in the context of large-scale health surveys entailing lengthy interviews. They also noted that older adults may struggle to remember all response options if interviewers read out all of them at once.

### Suggestions for improving EQ-5D IA data collection

In the last part of the FG discussion, participants shared thoughts and ideas on ways to improve EQ-5D data collection in large-scale surveys. We asked their view about moving EQ-5D questions to the beginning of the lengthy questionnaire. FG participants shared that they would ask these questions more carefully both because they have no prior information about the respondents and that respondents would be more attentive. However, all participants were candid in telling us that they might follow IA guidance initially but would likely develop time-saving adaptions once they became more familiar with the overall survey (i.e., all questions asked in the population health survey).

Two other suggestions were proposed by FG participants: (1) using visual aids, e.g. a hard copy of EQ VAS or use of a ‘smiley faces’ to indicate the levels of problems, to help respondents to understand and answer the questions; and (2) providing standardised examples to explain dimensions and levels.

## Discussion

EQ-5D data provide important population health insights globally. In China, concerns exist about the characteristics of EQ-5D data collected from population health surveys. Our study is the first to investigate face-to-face interviewer-administration of EQ-5D in Chinese population health surveys. We identified six ‘non-standard’ interview approaches to collecting EQ-5D data and three principal reasons underlying these approaches. Although interviewers recognised the advantages of the official IA version over the self-completion version, concerns were raised regarding its feasibility in large-scale population health surveys.

The prevalence of ‘non-standard’ interview approaches was high. Although self-completion versions were used in the survey and interviewers had not seen the IA version and notes, several observed approaches were unexpected, e.g. skipping questions, altering question and response wording. Both respondent characteristics and interviewers’ knowledge of the EQ-5D may influence interview style. In addition, survey-related factors such as overall length of the survey form and positioning of the HRQoL questionnaire may affect respondents’ patience and, in turn, prompt interviewers to adopt coping strategies, e.g. combining or skipping questions. As illustrated in Fig. 1, these factors may act individually or in combination to give rise to the observed non-standard approaches.

The ceiling effect observed for the EQ-5D-3L in 2020 survey (79.6%) was comparable to those reported in the National Health Service Survey (over 80%) [4, 6, 7], and higher than those reported in many other international studies [2]. While ceiling effects are a key concern in EQ-5D data, they represent only one of several potential consequences of non-standard interviewer administration, which may also affect other components of the instrument, including EQ VAS. Some interviewer approaches we observed may contribute to broader data quality issues. For example, skipping asking questions or focusing only on observable problems could result in over-reporting of ‘no problems’ and omission of mild issues. Leading questions, particularly suggesting no problems in dimensions, could also contribute to higher ceiling effects, as respondents are more likely to agree than disagree due to the ‘yes-saying’ tendency [19]. Similar issues were observed in the administration of the EQ VAS. Interviewers often suggested values, provided ranges, or interpreted responses on behalf of respondents. The range of non-standard interviewer behaviours, combined with the absence of ‘standard’ approaches to interviewer administration, may undermine the interpretation of EQ-5D data as being ‘self-reported’, and influence the distribution and validity of EQ-5D profile and EQ VAS responses.

On the other hand, some interviewer approaches may lead to more problems being reported, and therefore influence the distribution of EQ-5D responses, potentially reducing observed ceiling effects. Well-trained interviewers are expected to collect data efficiently while maintaining survey quality [19, 20]. The quality control mindset of interviewers may influence the responses ultimately recorded by ‘cross-checking’ responses, e.g., with respondents’ answers to similar questions preceding the EQ-5D, and their observations as physicians. Some studies have suggested that social desirability may affect face-to-face interviews [21], leading to under-reporting of problems, particularly in Asian countries [12, 22]. The role of interviewers may partly offset this tendency in some circumstances—because respondents had done health checks and previous questions had already identified their health problems. Nevertheless, when collecting self-reported HRQoL data, interviewers should not act as proxies for respondents or modify respondents’ answers.

Our findings highlighted three interrelated challenges in balancing practicality with accuracy when administering EQ-5D as part of lengthy interviews. These include respondent characteristics, interviewer knowledge and training, and survey structure. First, comprehension challenges among older adults and individuals with low literacy were prominent. In the 2020 full sample, nearly one-third of respondents had no formal education or only primary education. One of the reasons for ‘non-standard’ interview approaches was poor comprehension of EQ-5D questionnaire. This is consistent with evidence that older adults require explanations and clarification of EQ-5D descriptive system and EQ VAS questions [18] and that Asian populations had various interpretations of the EQ VAS [17]. In addition, wording such as ‘anxiety’ and ‘depression’ may not be commonly used in everyday language in rural areas and are difficult for these respondents to understand [12].

Second, interviewer knowledge of the EQ-5D and training are important considerations. Interviewer training should emphasise that the EQ-5D is intended to capture individuals’ subjective assessments of their own health, and that interviewers should not act as proxies. This is particularly important for interviewers with clinical backgrounds, who may be more accustomed to clinical assessment of health status. It is also essential to emphasise that the EQ-5D is a standardised instrument, and that its wording and order should not be modified. On the other hand, interviewer feedback suggests that IA guidance may need adaptation for large-scale interviewer-administered surveys. For example, the IA version suggests that interviewers read all response options ‘in one breath’, which may be cognitively demanding for respondents, requiring comprehension, recall of information on their health, mapping the retrieved information to the response options and retention of all options [19, 23]. This may explain the interviewers’ preference for a two-step approach, i.e. first asking whether any problems (yes or no), followed by severity levels. Similar approaches have been used in one study in China to reduce short-term memory load because most of the respondents were illiterate or with poor vision [24]. More broadly, not all HRQoL instruments have validated interviewer-administered versions or clear guidance for their use in interviewer-led surveys. When instruments designed for self-completion are administered by interviewers, this may contribute to variation in how questions are asked and responses are recorded.

Third, balancing interview length with respondent burden is a common challenge in large-scale studies [20, 25, 26]. Survey fatigue may introduce bias, reduce data quality or increase survey attrition [27, 28]. In this context, the position of the EQ-5D within a lengthy questionnaire, rather than questionnaire length alone, may influence interviewer behaviour and respondent engagement, although this requires further empirical investigation. While EQ-5D provides valuable information for monitoring population health [29, 30] and is relatively concise compared with other generic HRQoL instruments, its usefulness depends on appropriate administration.

The hypothesis that using interviewer-administered mode to collect EQ-5D data contributes to the high ceiling effects in population health survey in China has previously been noted [4]; however, empirical evidence testing this hypothesis and examining how interview approaches may influence EQ-5D profile data and EQ VAS more broadly remains limited. While this study did not empirically quantify the impact of interviewer behaviours on EQ-5D outcomes, it identified a range of non-standard administration practices and suggested plausible mechanisms through which these deviations may influence response patterns. Given the widespread use of face-to-face interviews to collect EQ-5D data in LMICs [2, 3], our results have broader implications beyond the Chinese context. Being aware of these issues and understanding their underlying reasons is the first step to strengthening data collection. Interviewer training on the principles of self-reported health and standardised EQ-5D administration may improve the consistency of data collection [31]. Other strategies, such as using visual aids or a two-step questioning approach to support respondent comprehension, may also be helpful in some populations [32]. However, the effects of these strategies on the validity, comparability, and overall quality of EQ-5D data require empirical evaluation before they can be recommended for routine implementation. In our ongoing research, we are evaluating two strategies within the 2023 Nanjing CDC survey: (1) providing interviewer training on EQ-5D; and (2) repositioning the EQ-5D questionnaire to the beginning of the survey. Using a quasi-experimental design with intervention and comparison sites, this study will assess the individual and joint effects of the two interventions on interviewer behaviour and EQ-5D data characteristics. Results from this work will be reported separately.

It is also important to clarify that this study focused on the data collection process and how it may influence EQ-5D responses and observed ceiling effects. Other factors may also affect how individuals perceive, interpret and report health problems. These include cultural and socioeconomic influences on subjective reporting of HRQoL, and individual factors such as literacy and health self-efficacy [33, 34]. These broader factors are beyond the scope of the current study but may interact with interviewer behaviours [34].

Nevertheless, there are several caveats. First, we analysed recordings of the EQ-5D-3L data collection process in 2020 whereas the FG participants discussed their experience with EQ-5D-5L in the 2021 survey. The non-standard interviewer behaviours identified in the 2020 recordings were also reported in the focus groups, suggesting that similar issues may arise when administering the EQ-5D-5L. However, the nature and extent of these issues may differ between the two instruments. For example, the greater number of response levels in the EQ-5D-5L may increase cognitive and response burden, which could present additional challenges for both respondents and interviewers. These differences should be considered when interpreting the findings. Second, the FGs used a convenience sample of interviewers recruited through Nanjing CDC. Third, we ran the FGs online with all participants joining as a group in-person. While this was the only feasible way at the time of this research (due to COVID-19 and travel restrictions), the articulation, data richness and interaction patterns may not be the same if conducted face-to-face [35–37]. Fourth, in the context of the municipal adult chronic condition surveillance survey, which is part of routine public health work conducted by local PHCs in China, interviewers were required to have a medical or public health background. The underlying reasons identified in this study, such as viewing the EQ-5D as an observer-assessed health measure, may therefore not be generalisable to surveys that employ other types of interviewers (e.g. higher degree research students) [5]. Lastly, while we identified a range of interviewer approaches and plausible pathways through which non-standard administration may influence responses, we were unable to determine the extent to which these behaviours affect ceiling effects or overall data quality. Future studies that systematically examine interviewer approaches within each interview, and their association with the probability of reporting full health while controlling for potential confounders, may provide empirical evidence on how interviewer behaviour contributes to ceiling effects.

## Conclusion

This study examined interviewer administration of the EQ-5D in a large-scale population survey in China. Non-standard interviewer behaviours were common; no interviews fully adhered to standard administration guidance. We identified potential reasons and plausible pathways through which non-standard administration may influence responses, and potential solutions to address them, including the need for interviewer training on the purpose of self-reported health and EQ-5D data collection. While findings are based on a single survey and should be interpreted with caution, they highlight important considerations for interviewer-administered EQ-5D data collection in similar survey contexts in China and other LMICs.

## Supplementary Information

Below is the link to the electronic supplementary material.

Supplementary Material 1

## Data Availability

The data that support the findings of this study are available from authors upon reasonable request.

## References

[1] Johnson, J. A., et al. (2025). EuroQol data for assessment of population health needs and instrument evaluation (EQ-DAPHNIE): A study for enhancing population health assessment. *Quality of Life Research*. 10.1007/s11136-025-03983-2 40317454 PMC12689827

[2] Cheng, L. J., et al. (2024). The ceiling effects of EQ-5D-3L and 5L in general population health surveys: A systematic review and meta-analysis. *Value in Health*. 10.1016/j.jval.2024.02.018 38467187

[3] Espinosa, O., et al. (2024). Estimation of societal values of health states preferences at the national level for low- and middle-income countries. *Value in Health Regional Issues,**39*, 40–48. 37976776 10.1016/j.vhri.2023.07.004

[4] Sun, S., et al. (2011). Population health status in China: EQ-5D results, by age, sex and socio-economic status, from the national health services survey 2008. *Quality of Life Research,**20*(3), 309–320. 21042861 10.1007/s11136-010-9762-x PMC3052443

[5] Yao, Q., et al. (2024). EQ-5D-5L population scores in mainland China: Results from a nationally representative survey 2021. *Value in Health,**27*(11), 1573–1584. 38977191 10.1016/j.jval.2024.06.012

[6] Yao, Q., et al. (2021). Population norms for the EQ-5D-3L in China derived from the 2013 national health services survey. *Journal of Global Health,**11*, 08001. 33692898 10.7189/jogh.11.08001 PMC7916444

[7] Lei, S., et al. (2017). Establishing benchmark EQ-5D-3L population health state utilities and identifying their correlates in Gansu Province. *China. Qual Life Res,**26*, 3049–3058. 28593532 10.1007/s11136-017-1614-5

[8] Li, D.-L., et al. (2024). EQ-5D-5L population norms for China derived from a national health survey. *Value in Health,**27*(8), 1108–1120. 38677363 10.1016/j.jval.2024.04.014

[9] Li, M., et al. (2021). Culture-related health disparities in quality of life: Assessment of instrument dimensions among Chinese. *Frontiers in Public Health,**9*, Article 663904. 34178922 10.3389/fpubh.2021.663904 PMC8221419

[10] Rowsell, A., et al. (2022). Systematic review of health-related quality of life (HRQoL) issues associated with gastric cancer: Capturing cross-cultural differences. *Gastric Cancer,**25*(4), 665–677. 35689705 10.1007/s10120-022-01309-6 PMC9225973

[11] Yip, K.-S. (2005). Chinese concepts of mental health: Cultural implications for social work practice. *International Social Work,**48*(4), 391–407.

[12] Yang, F., et al. (2020). Do rural residents in China understand EQ-5D-5L as intended? Evidence from a qualitative study. *PharmacoEconomics: Open*. 10.1007/s41669-020-00212-z 32285402 PMC7895880

[13] Salomon, J. A., et al. (2011). Comparability of patient-reported health status: Multicountry analysis of EQ-5D responses in patients with type 2 diabetes. *Medical Care*. 10.1097/MLR.0b013e3182239489 21918400

[14] Tong, A., Sainsbury, P., & Craig, J. (2007). Consolidated criteria for reporting qualitative research (COREQ): A 32-item checklist for interviews and focus groups. *International Journal for Quality in Health Care,**19*(6), 349–357. 17872937 10.1093/intqhc/mzm042

[15] Devlin, N., & Brooks, R. (2017). EQ-5D and the EuroQol Group: Past, present and future. *Applied Health Economics and Health Policy,**15*(2), 127–137. 28194657 10.1007/s40258-017-0310-5 PMC5343080

[16] EuroQol Group. *EQ-5D-5L | Interviewer Administered version*. 2023; Available from: https://euroqol.org/eq-5d-instruments/eq-5d-5l-available-modes-of-administration/interview-administered-version/.

[17] Tan, R. L., et al. (2021). How do respondents interpret and view the EQ-VAS? A qualitative study of three Asian populations. *Patient,**14*(2), 283–293. 32944897 10.1007/s40271-020-00452-5

[18] Marten, O., Brand, L., & Greiner, W. (2022). Feasibility of the EQ-5D in the elderly population: A systematic review of the literature. *Quality of Life Research,**31*(6), 1621–1637. 34613597 10.1007/s11136-021-03007-9 PMC9098572

[19] Bowling, A. (2005). Mode of questionnaire administration can have serious effects on data quality. *Journal of Public Health,**27*(3), 281–291. 15870099 10.1093/pubmed/fdi031

[20] Sharma, R., et al. (2022). Survey implementation process and interviewer effects on skipping sequence of maternal and child health indicators from national family health survey: An application of cross-classified multilevel model. *SSM - Population Health,**19*, Article 101252. 36268137 10.1016/j.ssmph.2022.101252 PMC9576585

[21] Hanmer, J., Hays, R., & Fryback, D. (2007). Mode of administration is important in US national estimates of health-related quality of life. *Medical Care,**45*(12), 1171–1179. 18007167 10.1097/MLR.0b013e3181354828

[22] Johnson, T. P., & van de Vijver, F. J. R. (2003). Social desirability in cross-cultural research. In J. A. Harkness, F. J. R. van de Vijver, & P. P. Mohler (Eds.), *Cross-cultural survey methods* (pp. 195–204). Wiley.

[23] Tourangeau, R., Rips, L., & Rasinki, K. (2000). *The psychology of survey response*. Cambridge University Press.

[24] Liang, Z., et al. (2019). Health-related quality of life among rural men and women with hypertension: Assessment by the EQ-5D-5L in Jiangsu, China. *Quality of Life Research,**28*(8), 2069–2080. 30830645 10.1007/s11136-019-02139-3

[25] Barr, M., *Quality issues in multipurpose ongoing population health surveys* in *School of Mathematics and Applied Statistics,* 2016, University of Wollongong,: Wollongong, Australia.

[26] Clark, R. G., Templeton, R., & McNicholas, A. (2013). Developing the design of a continuous national health survey for New Zealand. *Population Health Metrics,**11*(1), 25. 24364838 10.1186/1478-7954-11-25 PMC3880001

[27] Ghafourifard, M. (2024). Survey fatigue in questionnaire based research: The issues and solutions. *Journal of Caring Sciences,**13*(4), 214–215. 39974826 10.34172/jcs.33287 PMC11833437

[28] Jeong, D., et al. (2023). Exhaustive or exhausting? Evidence on respondent fatigue in long surveys. *Journal of Development Economics,**161*, Article 102992.

[29] Devlin, N., Parkin, D., & Janssen, B. (2020). Analysis of EQ-5D profiles. In N. Devlin, D. Parkin, & B. Janssen (Eds.), *Methods for analysing and reporting EQ-5D data* (pp. 23–49). Springer International Publishing. 33347096

[30] Janssen, B., & Szende, A. (2014). Chapter 3 population norms for the EQ-5D. In A. Szende, B. Janssen, & J. Cabses (Eds.), *Self-reported population health: An international perspective based on EQ-5D. *Springer. 29787044

[31] Jones, R., et al. (2025). “If we ask, we must act”: Co-designing the implementation of the EQ-5D-Y-5L as a paediatric patient reported outcome measure in routine hospital outpatient care for kids to meaningfully impact clinical visits (P-PROM ROCK phase 2). *Quality of Life Research*. 10.1007/s11136-025-03996-x 40448866 PMC12274246

[32] Milte, R., et al. (2023). A scoping review of the use of visual tools and adapted easy-read approaches in quality-of-life instruments for adults. *Quality of Life Research*. 10.1007/s11136-023-03450-w 37344727 PMC10624740

[33] Ashing-Giwa, K. T. (2005). The contextual model of HRQoL: A paradigm for expanding the HRQoL framework. *Quality of Life Research,**14*(2), 297–307. 15892421 10.1007/s11136-004-0729-7

[34] Pan, T., Huang, Y, Luo, N., Mao, Z., Calvert, M., Wu, J. Shiroiwa, T., Herdman, M., Lubetkin, E., Mulhern, B., Devlin, N, *What explains differences in ceiling effects in EQ-5D and other HRQoL instruments between populations? An investigation into possible reasons, existing evidence, and future research directions and priorities* in *EuroQol Academy Meeting* 2024: Copenhagen, Denmark.

[35] Graffigna, G., & Bosio, A. C. (2006). The influence of setting on findings produced in qualitative health research: A comparison between face-to-face and online discussion groups about HIV/AIDS. *International Journal of Qualitative Methods,**5*(3), 55–76.

[36] Abrams, K. M., et al. (2014). Data richness trade-offs between face-to-face, online audiovisual, and online text-only focus groups. *Social Science Computer Review,**33*(1), 80–96.

[37] Nicholas, D. B., et al. (2010). Contrasting internet and face-to-face focus groups for children with chronic health conditions: Outcomes and participant experiences. *International Journal of Qualitative Methods,**9*(1), 105–121.

