# Supplementary material for: Challenges with interviewer administration of EQ-5D questionnaires in a large-scale population survey in China: a qualitative analysis
Source: Qual Life Res. 2026 Jul 30;35(9):252. doi: 10.1007/s11136-026-04351-4 (PMC13424218; doi:10.1007/s11136-026-04351-4)
Supplement: Supplementary file 1 — Supplementary Material 1 [file 11136_2026_4351_MOESM1_ESM.docx]

**ESM1 FGD Protocol (English version)**

1. Welcome and establish ground rules for the focus group
2. Overview to the research plan and explanation of aim of the session
3. Planned timetable for the day
4. Prompts/Questions

**A qualitative analysis to understand interviewers’ experience in collecting EQ-5D data in large-scale population surveys**

1. **Welcome and establish ground rules for the focus group**

Thank you for agreeing to be part of this workshop. We appreciate your willingness to participate.

Introductions:

Purpose of the focus group:

The focus of the workshop is using EQ-5D questionnaire in large-scale population health survey in China.

EQ-5D questionnaire contains five questions and a visual analogue scale to measure health status of individuals. It is designed for self-completion by participants, for example patients in healthcare centers and hospitals answer this short questionnaire then doctor can know how good or bad a patient feel and how a patient’s health status has changed. It also can be completed by general population online or in face-to-face or telephone interviews. As you may recall, EQ-5D-5L has been used in the Nanjing CDC Chronic Disease and Risk Factor Survey. You have seen or asked these questions to participants.

However, studies have found some difference in data collected between self-completion and face-to-face interviews in China and other countries. The reason why we are undertaking this focus groups is to understand interviewers’ experience in using EQ-5D questionnaire in face-to-face interviews, challenges in the interview process and how the data collection procedure in large scale population survey can be improved in China.We need your input and want you to share your experience or observations and open thoughts with us.

[present a screenshot of EQ-5D-5L questions in tablet ]

Ground Rules

1. YOU ARE ALL EXPERTS

You have all be invited to participate because of your experience in this. We want everyone present to have an equal opportunity to express an opinion, irrespective of background, training and experience.

1. WE WANT YOU TO DO THE TALKING.

We would like everyone to participate so we may call on you if we haven't heard from you in a while.

1. THERE ARE NO RIGHT OR WRONG ANSWERS

Every person's experiences and opinions are important. Speak up and listen whether you agree or disagree. We want to hear a wide range of opinions and experiences. Every response is valued and there are no wrong answers. Differences of opinion are bound to occur and should be respected.

1. RESPECT

We want people to feel comfortable sharing ideas, experiences and views. So, we need to respect each other’s privacy and not repeat what is said here outside of the group.

1. WE WILL BE RECORDING THE SESSION

For our research we want to capture the discussion, but we won’t be identify anyone by name in our findings. You will remain anonymous. We will just identify general themes and key quotes.

**Any questions before we get started?**

1. **Overview to the focus group agenda and aim of each session**

Our aim today is to hear your experience and thoughts of using EQ-5D questionnaires in large scale population health survey, in either positive or negative way. The findings from this focus group will help us improve the data collection procedure in large scale population survey.

The workshop consists of two sessions.

**Session One** is to understand your own experience as an interviewer in the recent 2021 Nanjing CDC Survey, including interview settings, interview process, and any thoughts you have. Example questions include:

- Where were the interviews conducted? How was the environment/setting like?
- What was your experience asking these questions?
- What do you think is the most challenging part in the interview process?

After the first session, we will provide more background on EQ-5D questionnaire, e.g. how EQ-5D data is summarized, how EQ-5D data can be used, and introduce a new way for EQ-5D data collection (i.e. using interviewer administered version and general guidance).

Then in **Session Two**, we will invite you to share your thoughts and feedback on the interviewer administered version. Example questions include:

- Do you think the suggested way of conducting EQ-5D questions is feasible in large scale survey in China?
- What (else) you can think of for the EQ-5D data collection process in large scale population survey.

Prompts may be given in the middle of the discussion to ensure coverage of different interviewer-led patterns, which is identified from literature or our previous work on interviewer-led mode.

1. **Planned timetable for the day (2 hours)**

2:00 – 2:20 Introduction, ground rules, research aim and consent

2:20 – 3:05 Session One – Interview Experience

3:05 - 3:15 Information on EQ-5D

3:15- 3:55 Session Two – Thoughts on conducting EQ-5D interviews in large scale population survey

3:55 - 4:00 Close

Thank you for your time today. We will develop a summary of the discussion and conclusions from today’s focus group and send out to you in the next few weeks

**Prompts/Questions**– This section is for our own use only; not to share with participants before the focus group

**Session One**

1. Where were the interviews conducted? (Asked in Day 1 only)
2. What was the environment/setting like, quiet or a bit noisy, can take time to complete the survey or in a hurry? (Asked in Day 1 only)
3. **How do you usually ask these questions?/** **could you show me how did you usually ask these questions, assuming I am the participants（everyone show how they ask question in turn.** (Asked in both day)
   - - If some raises/mentions “skipping questions, combine a few questions together etc” we ask follow up questions:
     - We are interested in the way you ask these questions – what was the main reason you ask questions in this way/main reason you just chose to ask a few questions, why skipped xx dimension?
     - If no one shares ‘non standard MOA，We may use prompts here to coverage other observed interviewer-led patterns:
     - We have found some patterns from the literature, for example, interviewers sometimes combining a few of the five questions into one, skipping a few questions, or a situation when interviewer asks a question but receives no response… This is not uncommon in large scale survey. Does anyone have or observe any experience of these similar pattern?
     - If no one reads out the five levels, we ask follow up questions, after you asking these questions, how did participants respond? If their answer was not included in the five levels, what did you usually do?
     - Are there any different ways of asking those question you notice when your colleagues asking them?
4. How have you found the participant’s understanding and response? Do you have any difficulties in getting their answer? (Asked in Day 1 only)
5. How have you found the experience of using tablet (asking EQ-5D questions)? (Asked in Day 1 only)
6. Reflecting on your experiences (both good and bad), what do you think is the most challenging part in the interview process (including F2F interview, using tablet, organization)? (Asked in Day 1 only)
7. How long was it usually take to complete the EQ-5D questionnaire? (Asked in Day 2 only)
8. Any circumstances where you have to choose options for respondents? Please elaborate. (Asked in Day 2 only)

**Introduction on EQ-5D and suggested way of asking questions [prepare slides]**

- What information can we get from EQ-5D/ Why EQ-5D is useful

The responses to EQ-5D descriptive system we obtain from participants can be summarized as a 5-digit health state profile, that represents the level of reported problems on each of the five dimensions of health, e.g. 11223. EQ-5D health states may subsequently be converted into a single summary number (index value), which reflects how good or bad a health state is according to the preferences of the general population of a country/region.

Reporting descriptive statistics on patient reported outcomes (PRO) data can be very insightful. They can help identify which aspects of patient or population health have been most affected by their condition, or improved by treatment.

Information from VAS can be used as a quantitative measure of health outcome as judged by the individual respondents.

- How EQ-5D is suggested to be collected in face-to-face interview

“It is suggested that the interviewer follows the script of the EQ-5D. Although allowance should be made for the interviewer’s particular style of speaking, the wording of the questionnaire instructions should be followed as closely as possible. In the case of the EQ-5D descriptive system, the precise wording must be followed”

[**screenshot of two versions; user guidance from IA version]**

**Session Two**

1. As you may be aware, there are some differences in self-completion version (which was used in Nanjing CDC Survey) and the interviewer administered version. How have you found these two versions, considering that we will use them in the Survey and you will ask the questions. Which version you prefer, and why? Will either version change the way you asking questions? (Asked in both day)
2. Do you think the suggested way of conducting EQ-5D questions (i.e. using IA version and follow the guidance) is feasible? Any circumstances you may choose to follow all the guidance and text? (Asked in both days)
3. Did you receive any training before the survey? What kind of training you think may be helpful to support you conduct the survey? (Asked in both days)
4. What (else) we can do to improve the EQ-5D data collection process in large scale population survey – to make it more efficient. (Asked in both days)
5. If EQ-5D Qs are at the beginning of the questionnaire, would you think it will make a difference how you conduct the interview/ask EQ-5D questions? (Asked in both days)

Final question,

- Any other thoughts on the use of EQ-5D questions in large scale population survey, how we can improve the experience, or any thing you would like to share?

ESM 2 Appendix Table

| Table S1 Recording sample characteristics and non-standard interviewing approach identified | | | | | | | | | | | | | | | | | | | |  |
| --- | --- | --- | --- | --- | --- | --- | --- | --- | --- | --- | --- | --- | --- | --- | --- | --- | --- | --- | --- | --- |
|  | Sample characteristics | | | | | | | | Non-standard interviewing approach to EQ-5D descriptive system | | | | | | | Non-standard interviewing approach to EQ VAS | | | |  |
| ID | District | PHC | Age | Gender | education | Hukou | EQ-5D-3L profile | EQ VAS | Skip some questions | Alter question wording | Combine  questions | Alter  response options | Choose response based on own interpretation | Not asking any question | Self  Complete | Change wording | marking scores based on own interpret-ation | Skip asking | Self  Complete |  |
| 1 | A | A01 | 69 | F | Junior secondary | Urban | 11111 | 90 | MO | X | X | X | X |  |  | X | X |  |  |  |
| 2 | A | A01 | 51 | M | Junior secondary | Rural | 11111 | 60 |  |  |  |  |  |  | X |  |  |  | X |  |
| 3 | A | A02 | 68 | F | Junior secondary | Urban | 11122 | 20 |  |  |  |  |  | X |  |  |  | X |  |  |
| 4 | A | A02 | 54 | F | Senior high | Urban | 11111 | 80 |  |  |  |  |  |  | X |  |  |  | X |  |
| 5 | A | A03 | 70 | M | Primary | Urban | 11111 | 80 | MO SC AD | X |  | X |  |  |  |  |  | X |  |  |
| 6 | A | A03 | 44 | F | Senior high | Urban | 11111 | 90 | MO SC UA | X |  | X |  |  |  | X |  |  |  |  |
| 7 | A | A04 | 32 | M | Bachelor’s degree | Urban | 11111 | 85 |  |  |  |  |  |  | X |  |  |  | X |  |
| 8 | A | A04 | 64 | M | Senior high | Urban | 11111 | 80 |  |  |  |  |  |  | X |  |  |  | X |  |
| 9 | A | A05 | 52 | F | College diploma | Urban | 11111 | 100 | MO SC UA | X |  |  | X |  |  | X |  |  |  |  |
| 10 | A | A05 | 65 | M | Bachelor’s degree | Urban | 11121 | 80 | MO SC UA | X |  |  | X |  |  | X | X |  |  |  |
| 11 | A | A06 | 36 | F | Bachelor’s degree | Urban | 11111 | 90 |  |  |  |  |  |  | X |  |  |  | X |  |
| 12 | A | A06 | 67 | F | Junior secondary | Urban | 11122 | 60 | MO SC UA PD | X |  |  | X |  |  | X |  |  |  |  |
| 13 | A | A07 | 53 | M | Primary school | Rural | 11111 | 80 |  | X |  |  |  |  |  | X |  |  |  |  |
| 14 | A | A07 | 28 | F | Bachelor’s degree | Urban | 11122 | 80 |  |  |  |  |  | X |  |  | X | X |  |  |
| 15 | A | A08 | 49 | F | Junior secondary | Urban | 11111 | 80 |  | X | X |  |  |  |  | X |  |  |  |  |
| 16 | A | A08 | 51 | M | Junior secondary | Urban | 11111 | 80 | SC UA | X | X |  |  |  |  | X | X |  |  |  |
| 17 | B | B01 | 67 | M | Senior high | Rural | 11111 | 100 | MO SC UA PD | X |  |  |  |  |  |  | X | X |  |  |
| 18 | B | B01 | 32 | F | Senior high | Urban | 11111 | 80 | MO SC UA | X |  |  |  |  |  | X | X |  |  |  |
| 19 | B | B02 | 78 | F | No formal education | Rural | 22222 | 60 | SC UA PD | X |  |  | X |  |  | X | X |  |  |  |
| 20 | B | B02 | 55 | F | No formal education | Rural | 11111 | 80 | SC | X | X | X | X |  |  | X | X |  |  |  |
| 21 | B | B03 | 55 | F | Junior secondary | Urban | 11122 | 50 |  | X |  |  |  |  |  |  |  |  |  |  |
| 22 | B | B03 | 52 | F | Junior secondary | Rural | 11111 | 80 | MO SC UA PD | X |  |  |  |  |  | X |  |  |  |  |
| 23 | B | B04 | 65 | M | Primary school | Rural | 11111 | 70 | UA | X |  | X |  |  |  | X | X |  |  |  |
| 24 | B | B04 | 46 | F | No formal education | Rural | 11122 | 60 | MO SC UA | X |  | X | X |  |  | X | X |  |  |  |
| 25 | B | B05 | 31 | F | Senior high | Urban | 11111 | 100 | MO UA SC PD | X |  | X |  |  |  | X | X |  |  |  |
| 26 | B | B05 | 21 | F | Senior high | Urban | 11111 | 40 |  |  |  |  |  | X |  | X |  |  |  |  |
| 27 | B | B06 | 72 | F | No formal education | Rural | 11111 | 70 | SC UA PD AD | X |  | X |  |  |  | X | X |  |  |  |
| 28 | B | B06 | 66 | M | No formal education | Rural | 11121 | 60 | MO SC UA AD | X |  | X | X |  |  | X |  |  |  |  |
| 29 | B | B07 | 55 | F | Primary school (not completed) | Rural | 11111 | 70 | MO SC UA | X |  | X |  |  |  | X | X |  |  |  |
| 30 | B | B07 | 49 | M | Senior high school | Urban | 21121 | 80 |  | X |  | X |  |  |  | X |  |  |  |  |
| 31 | B | B08 | 49 | F | Junior secondary | Rural | 11111 | 70 | SC PD AD | X | X | X |  |  |  | X | X |  |  |  |
| 32 | B | B08 | 57 | F | Primary school | Rural | 11121 | 70 | UA AD | X | X | X | X |  |  |  |  | X |  |  |
| 33 | C | C01 | 43 | F | Bachelor’s degree | Urban | 11111 | 89 |  |  |  |  |  |  | X |  |  |  | X |  |
| 34 | C | C01 | 54 | F | Junior secondary | Urban | 11111 | 80 | MO SC AD | X | X |  |  |  |  | X | X |  |  |  |
| 35 | C | C02 | 31 | F | Senior high | Rural | 11111 | 80 |  |  |  |  |  | X |  | X | X |  |  |  |
| 36 | C | C02 | 59 | M | Senior high | Rural | 11111 | 70 |  | X |  |  |  |  |  | X | X |  |  |  |
| 37 | C | C03 | 50 | F | Junior secondary | Urban | 11111 | 80 |  | X |  | X | X |  |  | X | X |  |  |  |
| 38 | C | C03 | 39 | F | Junior secondary | Urban | 11111 | 80 | MO UA PD AD |  | X |  |  |  | X | X |  |  | 38 |  |
| 39 | C | C04 | 49 | M | Junior secondary | Rural | 11111 | 80 |  |  |  |  |  | X |  |  |  | X |  |  |
| 40 | C | C04 | 48 | F | Junior secondary | Urban | 11121 | 70 | UA AD | X | X |  | X |  |  | X |  |  |  |  |
| 41 | C | C05 | 72 | M | Junior secondary | Rural | 11111 | 80 | UA AD | X | X |  | X |  |  | X | X |  |  |  |
| 42 | C | C05 | 64 | M | Primary school | Urban | 11121 | 60 | MO UA PD AD | X |  |  |  |  |  | X | X |  |  |  |
| 43 | C | C06 | 57 | F | Junior secondary | Rural | 11122 | 70 | SC UA PD | X |  | X | X |  |  | X |  |  |  |  |
| 44 | C | C06 | 75 | M | Junior secondary | Rural | 11111 | 80 |  | X | X | X |  |  |  | X |  |  |  |  |
| 45 | C | C07 | 62 | M | No formal education | Rural | 11111 | 90 | UA | X | X | X | X |  |  | X | X |  |  |  |
| 46 | C | C07 | 49 | F | Primary school | Rural | 11111 | 80 |  | X | X |  |  |  |  | X |  |  |  |  |
| PHC: Primary Healthcare centres; Hukou: a system of household registration used in China; MO: mobility; SC: self-care; UA: Usual activities; PD: pain/discomfort; AD: anxiety/depression | | | | | | | | | | | | | | | | | | | | |
